# Bioequivalence study of fluticasone propionate nebuliser suspensions in healthy Chinese subjects

**DOI:** 10.3389/fphar.2024.1452596

**Published:** 2025-01-03

**Authors:** Feng Cheng, Tao Shen, Fucheng Zhang, Chenghao Lei, Ye Zhu, GuoJun Luo, Dawei Xiao

**Affiliations:** ^1^ Phase I Clinical Trial Site, Nanjing Gaoxin Hospital, Nanjing, Jiangsu, China; ^2^ Shanghai Chenpon Pharmaceutical Co., Ltd., Shanghai, China

**Keywords:** bioequivalence, safety, fluticasone propionate, asthma, healthy Chinese subjects

## Abstract

**Background:**

Fluticasone propionate is a synthetic trifluoro-substituted glucocorticoid, a highly selective glucocorticoid receptor agonist. Fluticasone propionate nebuliser suspensions is an inhaled corticosteroid with the low systemic bioavailability which provides a low risk (benefit outcome without the adverse effects that accompany systemically administered corticosteroids), referred as a first-line preventive agent for patients with persistent asthma. China has become one of the countries with the highest asthma mortality rate in the world in the past years. It urgently needs good generic drugs to help ease patients’ burden and improve their quality of life.

**Objective:**

The primary objective of this study was to evaluate the bioequivalence of fluticasone propionate nebuliser suspensions between test formulation (generic product) and reference formulation (original product, Flixotide Nebules^®^) with the pharmacokinetic parameters as the endpoint indicators and the secondary objective was to evaluate the safety of two inhalated fluticasone propionate nebuliser suspensions under the condition of fasting in healthy Chinese subjects.

**Methods:**

The bioequivalence study was conducted with a single-center, randomized, open-label, single-dose, two sequences, two-period crossover design. 24 healthy subjects were randomly assigned into T-R and R-T sequence groups with 12 patients in each group. The subjects were administered 1 mg (2 mL:0.5 mg,plastic ampoules) of generic fluticasone propionate nebuliser suspension as a test formulation or Flixotide Nebules^®^ as reference formulation and cross administration after sufficient washout period (5 days) for the second period study. The blood sample was collected at predetermined time points up to 48 h and the plasma concentration of fluticasone propionate was determined by HPLC-MS/MS in healthy subjects after inhalation of test or reference formulation. The non-compartment model method (NCA module) of the WinNonlin^®^ software (version 8.3) was used to calculate the pharmacokinetic parameters (C_max_, AUC_0-t,_ AUC_0-∞_) between the test formulation and the reference formulation were within the predefined range of 80.00% and 125.00%, bioequivalence of both formulations was demonstrated.

**Results:**

The 90% confidence intervals of the T/R ratio of the geometric mean of C_max_, AUC_0-t_, and AUC_0-∞_ for both formulations were 90.24%–112.68%, 96.99%–112.27% and 96.41%–111.59% respectively, which were all within the bioequivalent range of 80%–125%. No severe, suspicious or unexpected serious adverse reactions were reported.

**Conclusion:**

The test and reference formulations of fluticasone propionate nebuliser suspension were pharmacokinetic bioequivalent and were well tolerated and safe in all subjects.

## 1 Introduction

Asthma has become a global problem, one of the most common chronic diseases worldwide. The global prevalence, morbidity and mortality of asthma have increased sharply over the last 40 years (Braman, 2006; Serebrisky and Wiznia, 2019). It is estimated that approximately 300 million people worldwide currently have asthma, and will increase to 400 million by 2025 (Gulliver et al., 2007; Masoli et al., 2004). Asthma has a prevalence ranging from 1% to 18% (Al-Numani et al., 2015). The prevalence increases by 50% every decade, and approximately 180,000 deaths annually are attributable to asthma (Braman, 2006). It will cause more unemployment, fewer school days, poor quality of life, frequent emergency department visits, and hospitalizations of the patients (Al-Moamary et al., 2012).The economic burden of asthma in many countries is relatively high which may spend 1%–2% of their healthcare budget and it must be considered as the priority disorder in government health strategies (Serebrisky and Wiznia, 2019).

Asthma is a common chronic extensive airway inflammatory disorder which is characterized by airway hyper responsiveness, bronchial hyper-responsiveness, widespread but variable reversible and recurring airflow obstruction, lumen narrowing, airway remodeling and underlying inflammation (Gulliver et al., 2007; Al-Moamary et al., 2012; Hvizdos and Jarvis, 2000). The pathogenesis of asthma is very complex, and it is a multiple phenotypes syndrome (Expert Panel Working Group of the National Heart L et al., 2020) which is influenced by various inducers and provokers (Hvizdos and Jarvis, 2000). There are numerous cells and cellular elements (Braman, 2006) (such as eosinophils, mast cells, T lymphocytes, neutrophils, smooth muscle cells, airway epithelial cells, etc.) and cell components involved in the inflammation which induce Asthma (Crim et al., 2001). The main inducers and provokers are allergy. In addition, people with high airway reactivity and non-allergic stimulation may also cause airway contraction and finally induce asthma. These pathologic changes lead to recurring symptoms of wheezing, dyspnea, chest tightness and coughing. It often attacks or worsens at night and in the early morning (Braman, 2006; Gulliver et al., 2007). Most patients can relieve spontaneously or through treatment (Braman, 2006). After long-term standardized treatment and management, more than 80% of patients can achieve clinical asthma control.

Currently, the drugs used to treat asthma clinically can be divided into controller and reliever.Controller includes drugs that need to be used every day for a long time, including inhaled glucocorticoids (corticosteroids), systemic corticosteroids, leukotriene regulators, and long-acting drugs β 2-receptor agonists (LABA, which must be used together with inhaled corticosteroids), sustained-release theophylline, sodium tryptophan, anti-IgE antibodies and other drugs that help reduce the dose of systemic hormones. Reliever refers to drugs used on emergency as needed, including rapid inhalation β_2_-receptor agonists, systemic corticosteroids, inhaled anticholinergic drugs, short-acting theophylline and short-acting oral type β_2_-receptor agonists, etc. (Borghardt et al., 2018). These drugs relieve asthma symptoms by rapidly relieving bronchospasm. Inhaled corticosteroids are the most effective anti-inflammatory medication (Crim et al., 2001; Wood and Hill, 2009; Brutsche et al., 2000) and has been accepted as the first-line and cornerstone treatment for all individuals in the long-term control of persistent asthma since their introduction almost 50 years ago (Al-Numani et al., 2015; Hvizdos and Jarvis, 2000; Wood and Hill, 2009; Agertoft and Pedersen, 1994; Kirjavainen et al., 2018). Because the inhaled corticosteroids directly act on the respiratory tract by topical application, conferring high pulmonary drug concentrations and low systemic drug concentrations to exert a strong local anti-inflammatory effect, thus having a substantially better therapeutic index and safety than oral corticosteroids and other agents (Gulliver et al., 2007; Al-Numani et al., 2015; Hvizdos and Jarvis, 2000; Borghardt et al., 2018; Derendorf et al., 1998), most of which are inactivated by the liver after entering the blood through digestion and the respiratory tract, hence, fewer systemic adverse reactions happened (Brutsche et al., 2000). The previous studies show that inhaled corticosteroids can effectively reduce asthma symptoms, improve lung function, reduce airway hyperreactivity, reduce acute exacerbations of asthma, control airway inflammation, reduce the frequency and severity of asthma attacks, reduce mortality of asthma and improve quality of life (Al-Numani et al., 2015; Wood and Hill, 2009).

The exact mechanism of glucocorticoid in asthma inflammation is still unclear. Inflammation plays an important role in the pathogenesis of asthma. Glucocorticoids have been proven to have extensive inhibitory effects on allergic or non allergic inflammation by modulating multiple cell types (such as mast cells, eosinophils, basophils,neutrophils, macrophages and lymphocytes) and mediators (such as histamine, arachidonic acid and cytokines). The therapeutic effect of glucocorticoids on asthma may be attributed to their anti-inflammatory effect. On the molecular level, these anti-inflammatory actions are receptor-mediated, depending on the binding of the drug to the glucocorticoid receptor and subsequent transcriptional regulation of target genes (Crim et al., 2001). Recent data show that ICSs are well-tolerated, safe medications at the recommended dosages. ICSs act topically on lung epithelium to inhibit cell migration and activation and reduce airway hyperresponsiveness. ICSs block the late-phase (inflammatory) reaction to the allergen but not the early-phase (bronchospasm) reaction (Wood and Hill, 2009).

An ideal inhaled corticosteroid should demonstrate high pulmonary deposition and residency time (Gulliver et al., 2007) to have highly effective and lasting anti-inflammatory activity at the administration site (Möllmann et al., 1998), in addition to a low systemic bioavailability and rapid systemic clearance (Gulliver et al., 2007), which has only minimal systemic effects (Möllmann et al., 1998).Seven different ICS are currently available on the market for clinical use: fluticasone propionate, triamcinolone, budesonide, flunisolide, beclomethasone (beclometasone), mometasone, and ciclesonide (Gulliver et al., 2007). Fluticasone propionate (FP), the latest development in this group of inhaled corticosteroids is a synthetic trifluorinated corticosteroid with mainly androstane glucocorticoid activity (Kirjavainen et al., 2018) which is a highly selective, lipophilicitive and affinitive glucocorticoid receptor agonist (Crim et al., 2001; Derendorf et al., 1998). It mainly acts on the lungs and local respiratory tract. After inhalation of fluticasone propionate at the recommended dose, it shows strong and sustained anti-inflammatory effect in the lungs which is 18 times greater than dexamethasone (Al-Numani et al., 2015; Crim et al., 2001; Möllmann et al., 1998) with higher therapeutic index and efficacy (Brutsche et al., 2000), lower systemic effects than other inhaled corticosteroids (Gulliver et al., 2007; Brutsche et al., 2000; Möllmann et al., 1998; Thorsson et al., 1997) which can mitigate the symptoms and deterioration of asthma, prevent the decline of lung function, relieve acute exacerbations of asthma, reduce the risk of death from asthma, improve the control of asthma symptoms, reduce the use of other drugs, such as first-aid bronchodilators (Wood and Hill, 2009), Fluticasone propionate has a excessively high hepatic first-pass metabolism (Brutsche et al., 2000; Thorsson et al., 1997; Tony and Abdelrahim, 2022), very low oral bioavailability (Brutsche et al., 2000; Thorsson et al., 1997) (less than 1%) (Al-Numani et al., 2015; Möllmann et al., 1998; Tony and Abdelrahim, 2022), and 99% plasma protein bound (Al-Numani et al., 2015). It has a total blood clearance equivalent to hepatic blood flow (Thorsson et al., 1997). These indicate that following inhaled doses, any systemic activity results from the absorption of the drugs through the lungs, with a negligible contribution from the swallowed portion (Brutsche et al., 2000; Möllmann et al., 1998), therefore, has lower systemic exposure, thus make the incidence and severity of side effects significantly lower than other inhaled corticosteroids (Brutsche et al., 2000). Taken together, fluticasone propionate is one of the cornerstones and first-line in the treatment of moderate to severe asthma (Al-Numani et al., 2015; Crim et al., 2001; Brutsche et al., 2000), introducing generics of Fluticasone propionate products is essential, as the pricing of these medications remain a barrier to adequate patient care (Al-Numani et al., 2015).

Recently, a generic fluticasone propionate nebuliser suspension (test, T) has been developed by Shanghai Xin Huanghe Pharmaceutical Co., Ltd. in China. The primary objective of the present study was to evaluate the BE (bioequivalence) between the test formulation (T) and the reference formulation (R) of Fluticasone propionate in healthy Chinese subjects. Bioequivalence can be demonstrated if the 90% confidence interval of the geometric mean ratio of PK(pharmacokinetic) parameters (C_max_, AUC_0-t,_ AUC_0-∞_) between test and reference formulations are within the acceptable range of 80%–125%. The second objective was to evaluate the safety of a single dose of fluticasone propionate (2 mL:0.5 mg*2 plastic ampoules) in healthy Chinese subjects.

## 2 Methods

The study was conducted in accordance with the Declaration of Helsinki and the Good Clinical Practice (GCP). The study protocol, informed consent documents and advertisement,etc., were approved by the Ethics Committee of the Nanjing Gaoxin hospital (approval number: [2022]-012). Nanjing Gaoxin hospital Phase I Clinical Trial Site meets the satisfactory level of quality management system and bio-centre facility compliance, and obtains the quality management system qualification certificate (certificate NO:PMZ/QMS/2022/137). The site has been certified by the Pharmazone (the third-party certification authority), The rights and interests of subjects will be fully protected.Written informed consents (IC) were provided by all subjects prior to participating in any study-related activities in the study. Adequate time and opportunity were given to inquire about details of the study and to decide whether or not to participate before signing the IC.

### 2.1 Subjects

Twenty-four healthy male/female subjects (as determined by medical history, physical examination, vital signs, electrocardiogram, and laboratory tests at screening) aged 18–45 years with a body weight of male ≥50 Kg or females ≥45 Kg and BMI of 18.5–26.0 kg/m^2^ as well as promised that they would not have a fertility or sperm/ovum donation plan during the study period and within 60 days after the end of the study, and that they would voluntarily take one or more non drug contraceptive measures (such as complete abstinence, contraceptive ring, partner ligation, etc.) during the trial period were included. Subjects were excluded if they are allergic to fluticasone propionate or its analogues or prone to be allergic to multiple drugs or food or pollen; having respiratory diseases (active or static pulmonary tuberculosis, chronic bronchitis, emphysema, chronic obstructive pulmonary disease, asthma, chronic cough) and any other system or organic diseases or mental disorders; used to have smoking history within 1 year prior to screening or smoking test results are positive at screening; persons with any history of drug dependence or positive urine drug screening results; those who frequently drink alcohol; pregnant/lactating women or women of childbearing potential; on any prescription including vitamins and herbal supplements within 30 days prior to screening; on any inducers or inhibitors of hepatic metabolism CYP3A4 enzymes activity (such as inducers: barbiturates, carbamazepine, phenytoin, glucocorticoids, omeprazole; inhibitors: SSRI antidepressants, cimetidine, diltiazem, macrolides, nitroimidazoles, sedative hypnotics, verapamil, fluoroquinolones, antihistamines), etc, within 30 days prior to screening; participants in any clinical investigation within 3 months prior to screening or plan to participate in other clinical trials during the study; receiving major surgery within 3 months (90 days) prior to screening; losting/donating more than 400 mL of blood within 3 months (90 days) prior to screening; with HBsAg, anti-HCV, anti-HIV positives; clinically significant abnormalities in electrocardiogram, physical examination chest X-ray/CT examination laboratory tests and other situations determination by doctors or investigators.

### 2.2 Study design

**FIGURE 1:**
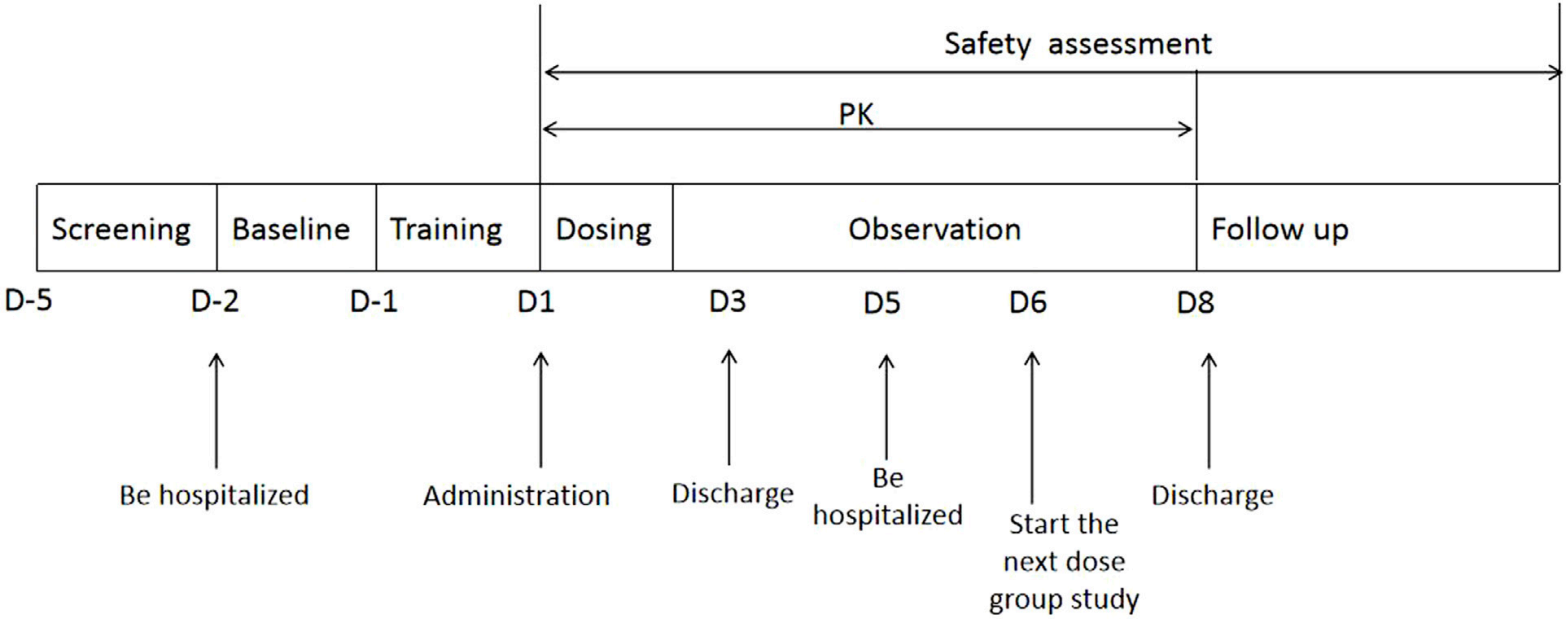
The flow chart of the experiment process of the study.

The flow chart of the experiment process is shown in Figure 1. We recruited twenty-four subjects and used a blocked randomization method with a 1: 1 ratio to randomize to T-R and R-T two sequence groups. Each group included 12 subjects, and adopted a randomized, open-label, two-period crossover design. Subjects were scheduled for dosing as per the randomization schedule in each period which is shown in Table 1.The test formulation and reference formulation of fluticasone propionate nebuliser suspension was inhalated for 12 min at the first period and then crossover next after a 5-day washout period. Test formulation (T): fluticasone propionate nebuliser suspension manufactured by Shanghai Xin Huanghe Pharmaceutical Co., Ltd., with specification of 2 mL: 0.5 mg (Batch No.: 11922003); Reference formulation (R): fluticasone propionate nebuliser suspension (Fluxotide Nebules ^®^) manufactured by GlaxoSmithKline Australia Pty Ltd. and provided by Shanghai Xin Huanghe Pharmaceutical Co., Ltd.; with specification of 2 mL: 0.5 mg (Batch No.: GM6873).

**TABLE 1:** Randomization schedule for the 24 subjects included in the study.

Subject	Sequence	Period I	Period II
K001	R-T	R	T
K002	R-T	R	T
K003	T-R	T	R
K004	R-T	R	T
K005	T-R	T	R
K006	T-R	T	R
K007	T-R	T	R
K008	R-T	R	T
K009	R-T	R	T
K010	R-T	R	T
K011	T-R	T	R
K012	T-R	T	R
K013	R-T	R	T
K014	T-R	T	R
K015	R-T	R	T
K016	T-R	T	R
K017	T-R	T	R
K018	R-T	R	T
K019	T-R	T	R
K020	R-T	R	T
K021	R-T	R	T
K022	T-R	T	R
K023	T-R	T	R
K024	R-T	R	T

All subjects were fasted for at least 10 h prior to each treatment period until 4 h after drug administration, and were forbidden to drink water before and within 1 h after administration, and did not strictly control the amount and time of drinking water in the rest of the time. The subjects could have a unified standard meal (low-fat light diet) for lunch and dinner after fasting phase. The recipes of the two treatment periods were the same, and the meal plan during the two periods should be consistent.

### 2.3 Pharmacokinetic analysis of blood samples

In each period, venous blood samples (4 mL) for analysis of plasma drug concentrations were collected in potassium EDTA (KEDTA) tubes pre-dose and at 5 min, 10 min, 15 min, 20 min, 25 min, 30 min, 40 min, 50 min, 1 h, 1.25 h, 1.5 h, 1.75 h, 2 h, 2.5 h, 3 h, 4 h, 6 h, 9 h, 12 h, 24 h, 36 h and 48 h after the start of dosing, gently reversed and mixed well and were placed in an ice bath for keeping before centrifugation. Within 120 min after the blood sample is collected, it enters the cryogenic centrifuge (preset at 4°C, 2500 g) for 5 min. After the centrifugation operation, the sample is taken out of the centrifuge, and the plasma is promptly packed in two labeled cryo vials. The amount of plasma in one cryo vial shall be at least 1.2 mL for analysis and test (test cryo vial); the remaining plasma is sub-packed in another cryo vial as a backup. The sub-packaged plasma can be placed in a refrigerator below −60°C for freezing until the cold chain is delivered to the sample analysis laboratory for pharmacokinetic analysis. Plasma samples were prepared by solid-phase extraction at room temperature and yellow light conditions; fluticasone propionate concentrations were determined by high performance liquid chromatography-tandem mass spectrometry (HPLC-MS/MS) assay with lower limit of quantitation (LLOQ) of 2.00 pg/mL (calibration range,2.00–600 pg/mL). Chromatographic retention times and peak areas were collected and processed by Watson LIMS (version 7.6.1, Thermo Fisher Corporation U.S.A.). All separations were carried out at 40°C using ACE Excel2 C18 50 × 21 mm. The mobile phase and flow rate are as follows: mobile phase A: 100% water containing 10 mM ammonium acetate and 0.1% formic acid; Mobile phase B: 100% acetonitrile with 0.1% formic acid; The flow rate is 0.600 mL/min. Adopting stepwise elution. And the mass spectrometer was operated in positive electrospray ionization mode. Identifications were based on multiple reaction monitoring transitions; m/z 501.2–293.2 for fluticasone propionate and m/z 506.2–293.2 for the standards of internal standard (IS) fluticasone propionate D_5_. Analytical data were processed using the Analyst 1.7.2, Applied Biosystems, U.S.A; Microsoft Office 2013, Microsoft, U.S.A; Watson LIMS 7.6.1, Thermo Fisher Corporation, U.S.A. The range of precision deviation between batches of precision range (CV) was below 15%.

### 2.4 Safety assessments

The physicians are responsible for observing any adverse event of all subjects during the clinical study (from receiving the test drug to the last follow-up) including clinical symptoms, physical examination, vital signs, laboratory tests and abnormalities in 12 lead ECG, for the safety evaluation in terms of NCI-CTCAE 5.0 standard. The clinical manifestations, severity, occurrence time, end time, treatment measures and outcomes were recorded, and the correlation between them and the investigational drug was determined.

### 2.5 Data analysis

Pharmacokinetic parameters of each subject after administration of test formulation and reference formulation were calculated using the non-compartmental model (NCA module), WinNonlin (Version 8.3). Primary PK parameters were C_max_ (peak concentration), AUC_0-∞_ (area under the curve from time zero to time infinity), AUC_0-t_ (area under the curve from time zero to time of the last measurable concentration of fluticasone propionate), wherein, C_max_ is expressed by the measured value, AUC_0-t_ is calculated by linear trapezoidal method, AUC_0-∞_ = AUC_0-t_ + C_t_/λ_Z_ (t is the sampling time of the last measurable blood drug concentration; C_t_ is the last measurable sample drug concentration; λ_Z_ is the terminal elimination rate constant obtained from the linear part at the end of the logarithmic concentration time curve. The best curve of the elimination phase is obtained by the least square method. Multiply the slope and 2.303 to obtain λ _Z_ value). Secondary PK parameters were T_max_ (time to the peak concentration), λ_z_ (Elimination rate constant), AUC__%Extrap_ (Percentage of AUC_0-∞_ due to extrapolation from T_last_ to infinity), and t_1/2_ (half elimination time). At the same time, calculate the arithmetic mean, standard deviation, coefficient of variation, median, quartile, maximum, minimum and geometric mean of each parameter. SAS Software (Version 9.4) is adopted for bioequivalence evaluation for statistical analysis. C_max,_ AUC_0-t_ and AUC_0-∞_ are logarithmically transformed and then subject to multifactor analysis of variance (ANOVA) to test the level α = 0.05. In the ANOVA model, sequence, formulation, and period are taken as fixed effects, and subjects (order) are taken as random effects to judge the significance of differences between drug formulations, individuals, periods, and administration order.When the 90% confidence interval of the geometric mean ratio of C_max_, AUC_0-t_ and AUC_0-∞_ of the test preparation and the reference formulations is within the range of 80.00%–125.00%, it is considered that the two formulations are bioequivalent.

## 3 Results

### 3.1 Characteristics of subjects in the study

**FIGURE 2:**
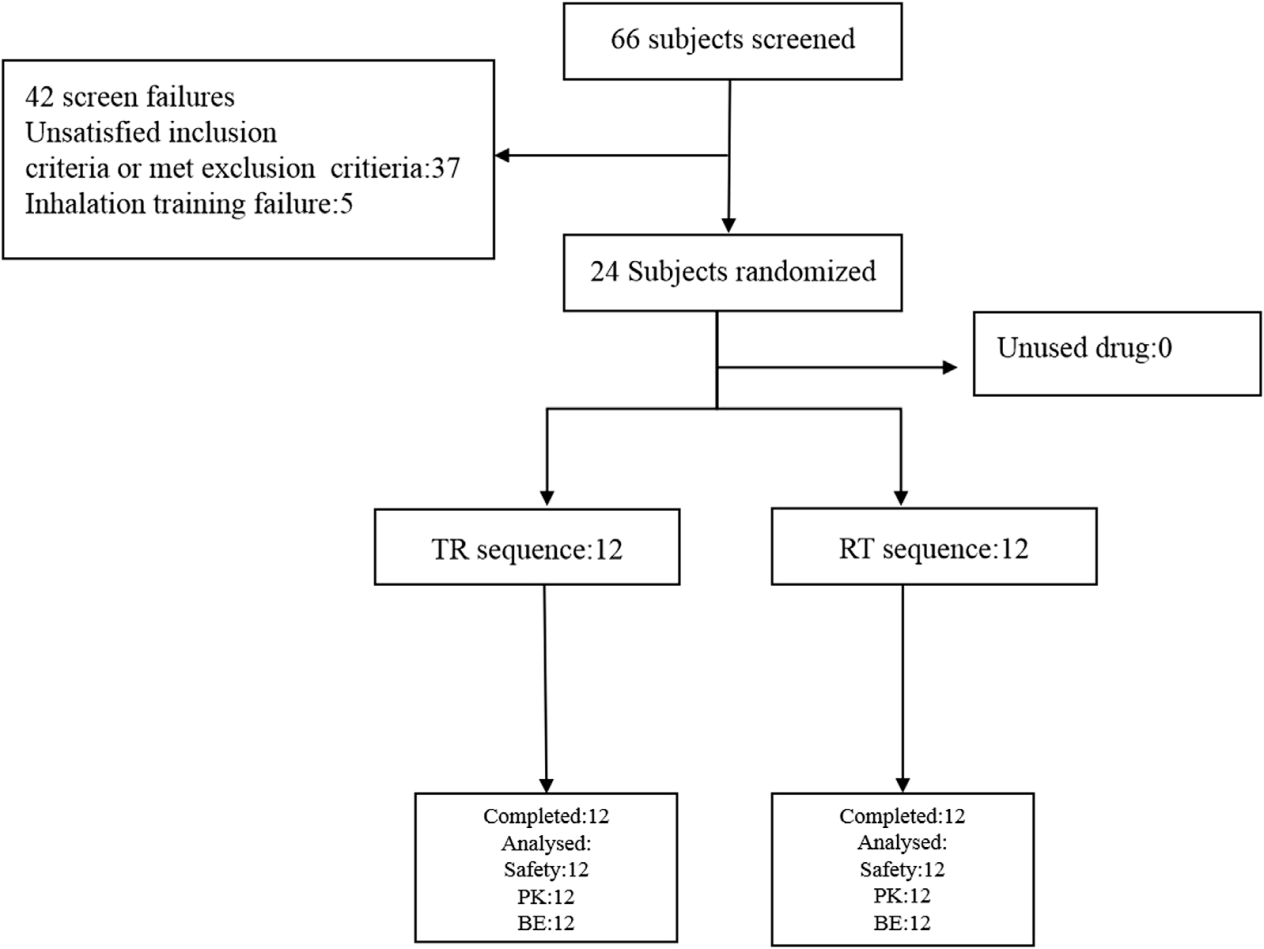
The scheme of the inclusion and exclusion process of the subjects.

A total of 24 healthy subjects were enrolled in the study, including 17 males and 7 females. The average age was 26.33 ± 5.74 years (range 18–45 years, inclusive). The average height is 167.59 ± 7.61 cm; The average weight is 63.14 ± 8.30 Kg. Among the 24 subjects, 23 were Han nationality and 1 was Zhuang nationality. The baseline demographics of the subjects in the study are shown in Table 2. The inclusion and exclusion process of the subjects in the study are shown in Figure2, and all the subjects completed the study successfully.

**TABLE 2:** Baseline demographics of subjects in the study.

	N (Nmiss)	Mean (SD)	Median (Q1, Q3)	Min,Max
Age (years)	24 (0)	26.33 (5.74)	26.50 (21.00,31.00)	19.00,36.00
Height (cm)	24 (0)	167.59 (7.61)	169.50 (163.50,171.50)	151.10,179.50
Weight (kg)	24 (0)	63.14 (8.30)	62.15 (58.75,68.40)	49.50,82.30
BMI(kg/m2)	24 (0)	22.40 (1.66)	21.75 (21.35,23.95)	20.00,25.60

BMI, body mass index; BMI , Weight/Height2; SD, standard deviation.

### 3.2 Bioequivalence analysis

**FIGURE 3:**
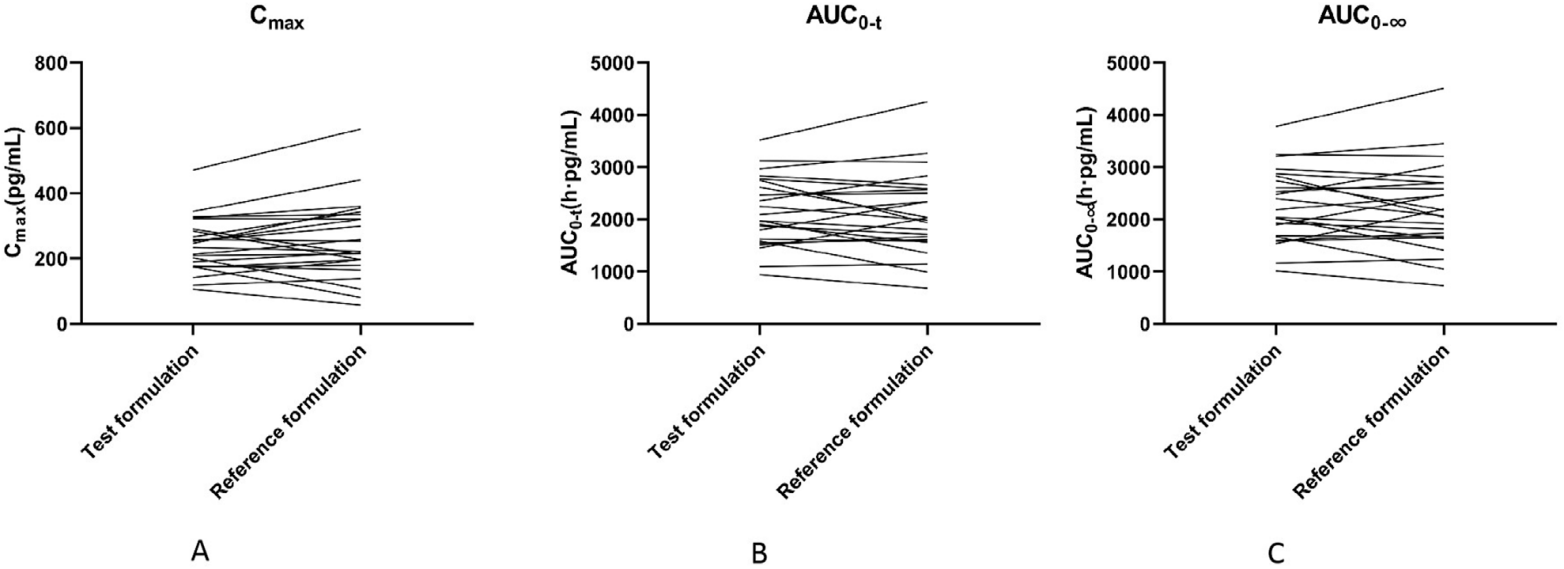
Comparison of individual C_max_ **(A)** panel,AUC_0-t_ **(B)** panel and AUC_0-∞_ **(C)** panel values for two fluticasone propionate formulations.

All subjects in the study were administrated successfully, and there was no need to reschedule any treatment period. The primary pharmacokinetic parameters of two fluticasone propionate formulations (test or reference) following a single dose of inhalated 1 mg were summarized in Table 3 and a comparison of the individual values of C_max_,AUC_0-t_ and AUC_0-∞_ is provided in Figure 3.

**TABLE 3:** Pharmacokinetic parameters of aerosol inhaled fluticasone propionate nebuliser suspension 1 mg for test formulation (T) and reference formulation (R).

Parameters (unit)	Mean ± SD (CV%) (N^#2^ = 24)	
	Test formulation (T)	References formulation (R)
T_max_ (h)^#1^	0.83 (0.41, 2.00)	0.66 (0.25, 3.99)
C_max_ (pg/mL)	239.83 ± 82.64	252.97 ± 120.06
AUC_0-t_ (pg·h/mL)	2146.59 ± 656.37	2107.33 ± 795.21
AUC_0-∞_ (pg·h/mL)	2250.07 ± 696.18	2220.49 ± 837.95
λ_z_ (1/h)	0.06 ± 0.01	0.06 ± 0.01
t_1/2_ (h)	11.09 ± 1.97	11.59 ± 1.39

#1: T_max_ represents the median (minimum, maximum).

#2: N represents the number of people in the PK, analysis set.

**FIGURE 4:**
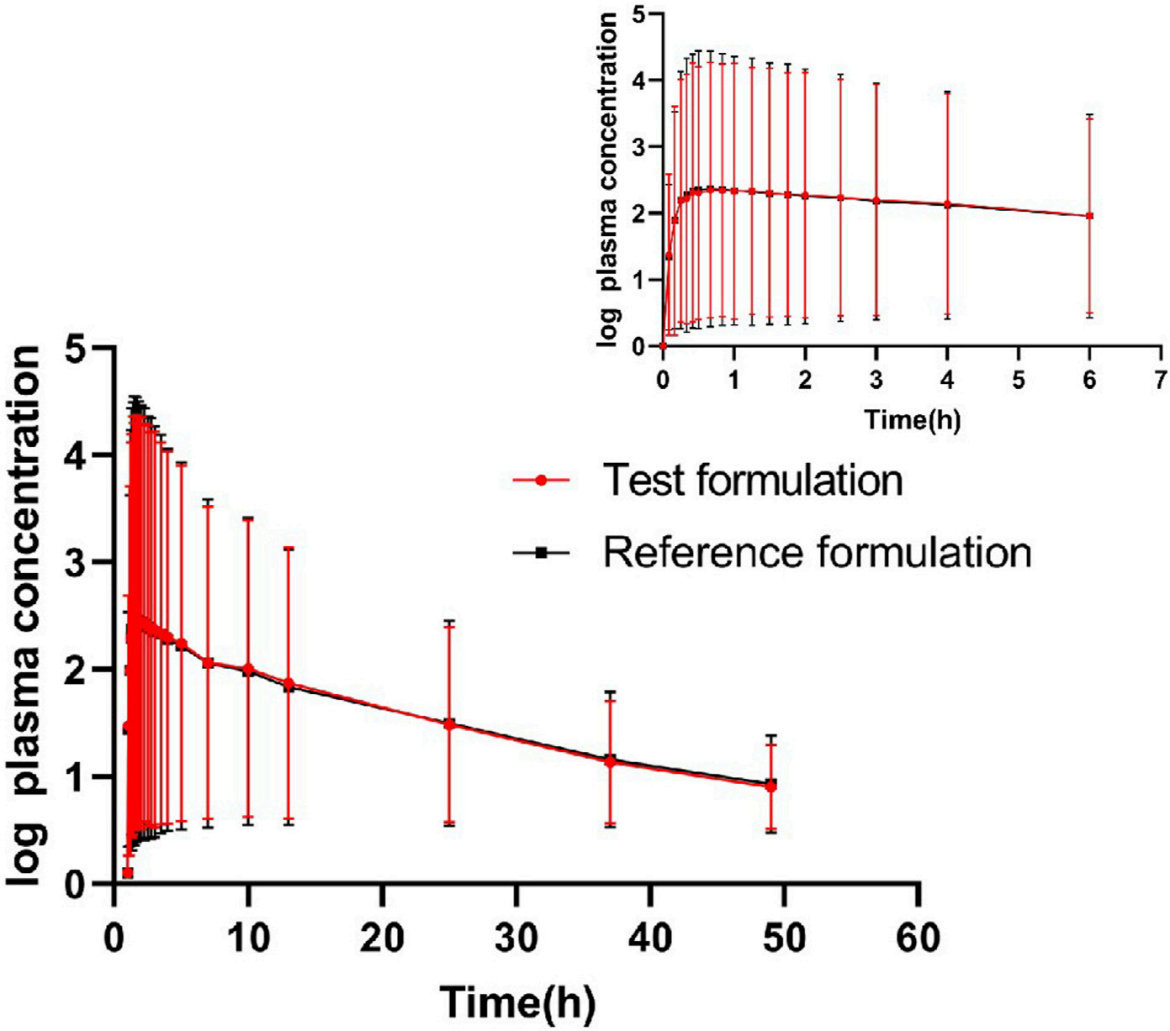
Logarithmic curve of fluticasone propionate concentration-time profiles after administration of single inhalation doses of fluticasone propionate 1 mg of the test and reference formulations.

**FIGURE 5:**
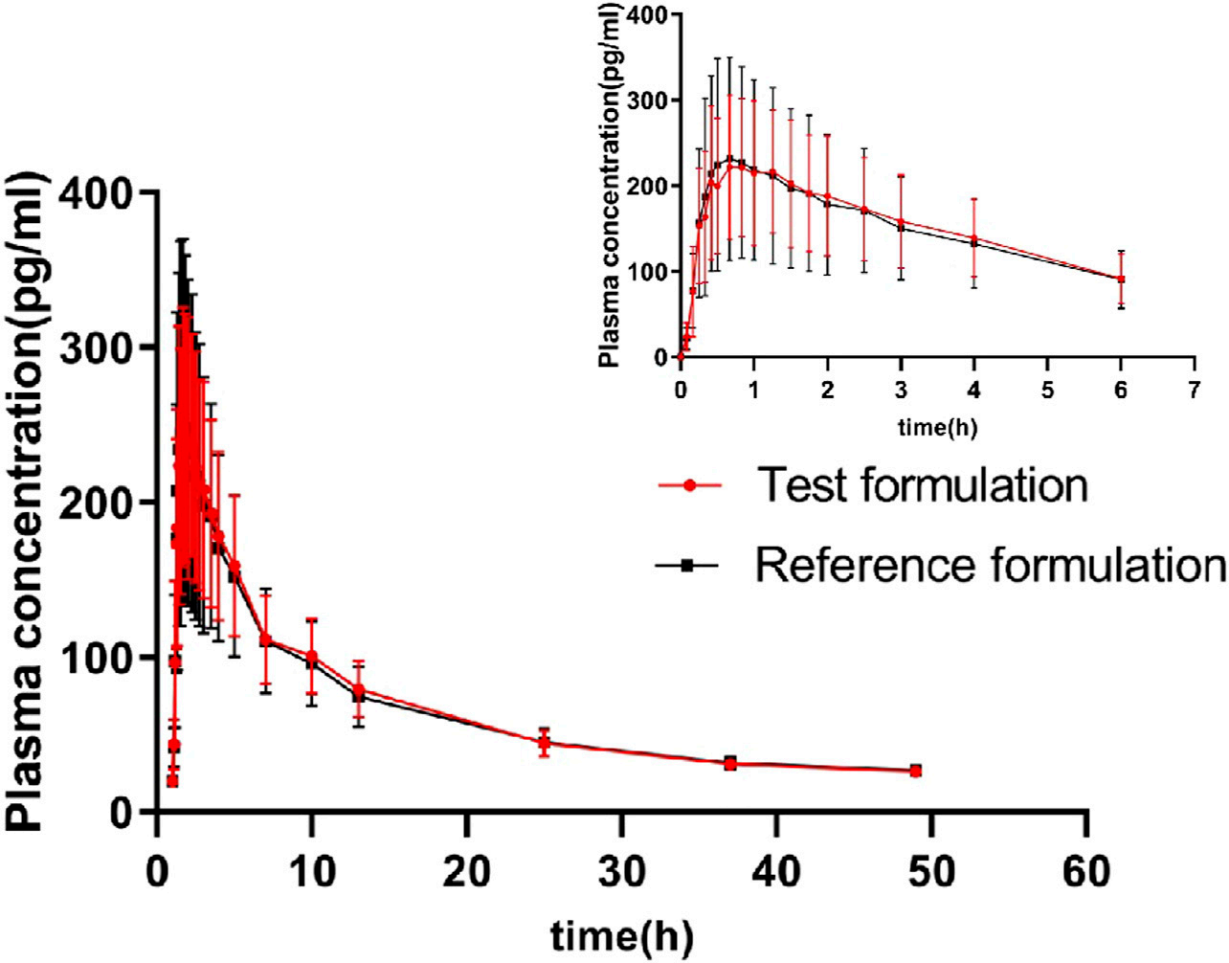
The concentration-time profiles of fluticasone propionate after administration of single inhalation doses of fluticasone propionate 1 mg of the test and reference formulations.

The geometric means of primary endpoints, C_max_, AUC_0-t_ and AUC_0-∞_ were 239.83 pg/mL, 2146.59 pg h/mL and 2250.07 pg h/mL for the test formulation and 252.97 pg/mL,2107.33 pg h/mL and 2220.49 pg h/mL for the reference formulation, respectively. The concentration-time profiles after inhalation of two fluticasone propionate formulations (test versus reference) were shown in Figures 4, 5. The plasma concentrations of fluticasone propionate appeared to be comparable between the test and reference formulation over the 48 h sampling period.

The pharmacokinetic parameters C_max_, AUC_0-t_, AUC_0-∞_ of fluticasone propionate were converted by natural logarithm and analyzed by multivariate ANOVA. The results showed no statistical difference in C_max_, AUC_0-t_ and AUC_0-∞_ between the test formulation and the reference formulation in the administration sequence, administration period and formulations.The results were showed in Table 4.

**TABLE 4:** Analysis of variance of the pharmacokinetic parameters.

Main factors	*p*-Value		
	Ln (C_max_) (pg/mL)	Ln (AUC_0-t_) (pg·h/mL)	Ln (AUC_0-∞_) (pg·h/mL)
Administration sequence	0.985	0.641	0.651
Administration period	0.855	0.489	0.598
formulation factor	0.899	0.328	0.400

After logarithmic transformation, the two-side *t*-test of PK parameters (C_max_, AUC_0-t_ and AUC_0-∞_) showed that the low side test *p* values were all smaller than 0.001; The *p* values of AUC_0-t_ and AUC_0-∞_ in the high side test were both <0.001, and the *p*-value of C_max_ was 0.002. By rejecting H_0_ and accepting H_1_, it can be considered that PK parameters (C_max_, AUC_0-t_ and AUC_0-∞_) of the test formulation and reference formulation in this test meet the bioequivalence standard. By calculating 90% confidence interval of the geometric mean ratio of PK parameters (C_max_, AUC_0-t_ and AUC_0-∞_) of test and reference formulation. The 90% confidence interval of C_max_ is 90.24%–112.68%; the 90% confidence interval of AUC_0-t_ is 96.99%–112.27%; the 90% confidence interval of AUC_0-∞_ is 96.41%–111.59%. PK parameters C_max_, AUC_0-t_ and AUC_0-∞_ are all in the range of 80.00%–125.00%, so it is considered that the test formulation is equivalent to the reference formulation. PK parameters of the primary end points are shown in Table 5.

**TABLE 5:** BE evaluation of aerosol inhaled fluticasone propionate suspension 1 mg for test formulation (T) and reference formulation (R).

Parameters (unit)	Corrected geometric mean and ratio (N^#1^ = 24)			Intra-individual variation of subjects%CV	90% CI	Power of test%
	Test formulation (T)	References formulation (R)	(T/R)%			
C_max_ (pg/mL)	226.47	224.59	100.84	22.69	(90.24, 112.68)	90.77
AUC_0-t_ (pg·h/mL)	2044.58	1959.33	104.35	14.84	(96.99, 112.27)	99.30
AUC_0-∞_(pg·h/mL)	2142.58	2065.73	103.72	14.83	(96.41, 111.59)	99.53

#1: N represents the number of subjects in the PK analysis set.

### 3.3 Safety evaluation

Among the 24 subjects who entered the safety analysis set, 1 case of adverse event (Urine ketone body positive) was observed after R formulation in the second period and got recovered without further treatment. The severity of the adverse event was Grade I (mild), and the relationship between the adverse event and the study formulation was probably unrelated. No other clinically significant abnormality was found in the laboratory examination results; no serious adverse event and no death occurred. The test results showed that both test formulation and reference formulation were safe. Summary of adverse events are shown in Table 6.

**TABLE 6:** Summary of adverse events.

	Test formulation (T)	References formulation (R)	Total on treatment^#1^
Number of participants, n (%)	24 (%)	24 (%)	24 (%)
Any AE,n (%)	0	1 (4.2)	1 (4.2)
Severe AEs, n (%)	0	0	0
Serious AEs, n (%)	0	0	0
AEs by system organ class, n (%)			
Investigations:Ketone bodies urine positive	0	1 (4.2)	1 (4.2)

#1: each participant was administered T or R and cross administration after sufficient washout period (5 days) for the second period study.

## 4 Discussion

Pulmonary administration is a challenging route of administration. Firstly, the efficacy of inhalation depends on the location of drug deposition in the lungs. The deposition of inhaled drugs is a complex process that depends on the anatomy and physiology of the lungs, the physicochemical properties of the drug, the properties and characteristics of the formulation, and the type of drug delivery device, etc. (Douroumis et al., 2012). In order to ensure the accuracy and consistency of drug delivery in this trial, it is very necessary to select trial participants who have no respiratory diseases and exclude those who have smoking history, oral ulcers, pharyngitis, etc. They also need to be trained for simulated drug delivery by aerosol inhalation of normal saline. The researcher performing drug delivery fully evaluates the drug inhalation behavior of the trial participants, including understanding, comprehension, compliance, and consistency of operational behavior, etc., and provides sufficient training before formal drug delivery to strengthen the cooperation and compliance of the trial participants, ensure relatively consistent inhalation frequency, depth and duration, and minimize intra-individual differences. In order to reduce the risk of cross-contamination among trial participants during inhalation, the entire administration process needs to be carried out in a negative pressure room that meets the requirements, wearing standardized isolation clothing, and strictly controlling factors such as the entire inhalation process and sample collection environment to avoid cross-contamination among trial participants. The Fluticasone propionate suspension for inhalation is a suspension of fine particles. The fine particles will settle after standing. It needs to be fully shaken before administration to avoid inaccurate dosage.

Fluticasone propionate has been formulated as an suspension for inhalation delivered directly to the lungs, and developed as an effective therapy to treat patients with moderate to severe asthma (Al-Numani et al., 2015; Crim et al., 2001; Brutsche et al., 2000).To assess pulmonary deposition after inhaled administration, absorption of the active substance from the GI tract must often be blocked with charcoal, whereas for total systemic exposure, absorption from both the lung and GI tract must be considered. Although most of the inhaled other drug dose remains in the mouth and will be absorbed from the GI tract, ultimately resulting in higher systemic exposure (Borghardt et al., 2018; Zou et al., 2020). However, Fluticasone propionate is absorbed only from the lungs, this indicates a relatively long pulmonary residence time at the site of action (Derendorf et al., 1998), and fluticasone propionate has excessive first pass effect, low systemic bioavailability (fluticasone has an oral bioavailability of <1% (Al-Numani et al., 2015; Crim et al., 2001; Brutsche et al., 2000; Derendorf et al., 1998; Möllmann et al., 1998; Thorsson et al., 1997; Tony and Abdelrahim, 2022) and 99% plasma protein bound (Al-Numani et al., 2015)) and rapid systemic clearance as introduced before (Gulliver et al., 2007; Brutsche et al., 2000; Thorsson et al., 1997; Tony and Abdelrahim, 2022) and it is estimated that the systemic bioavailability of fluticasone propionate nebuliser suspensions inhalation is 8% (referring as NMPA Guidelines for the bioequivalence study of oral inhaled fumulation),therefore, the amount fluticasone propionate swallowed after inhalation contributes minimally to systemic exposure, negligible exposure through GI tract has also been confirmed (Kirjavainen et al., 2018). Hence, systemic absorption of inhaled fluticasone propionate occurs mainly through the lungs and administration of charcoal for lung deposition comparisons is not needed. The relative deposition in the lungs of fluticasone propionate (FP) after inhalation is easily and precisely to be measured using plasma sampling with pharmacokinetic techniques (Tony and Abdelrahim, 2022), so we conducted this bioequivalence study without concomitant administration of charcoal. When two medications have identical pharmacokinetic and lung deposition patterns, they are considered bioequivalent (Al-Numani et al., 2015; Möllmann et al., 1998; Zou et al., 2020).Results shows that the C_max_ of fluticasone propionate after inhaled administration 1 mg is almost similar to that in published studies co-administrated with charcoal which is 0.26 ± 0.14 ng/mL (Möllmann et al., 1998).

In this study, we selected healthy subjects, which included healthy male and female volunteers, as the studies found in the literature suggest that BE testing of inhaled fluticasone propionate in healthy volunteers would be more sensitive than that in asthmatic patients because pharmacokinetic parameter values are higher in healthy volunteers (Brutsche et al., 2000; Kirjavainen et al., 2018) and variability which is not related to differences between the products is lower (Kirjavainen et al., 2018). As a consequence, studies with healthy volunteers allow the demonstration of equivalence with a smaller number of subjects and lesser exposure to an investigational medicinal product. The study was carried out with a single dose, open, and randomized crossover design. The dose was 1 mg of fluticasone propionate as recommended, such a dose enabled the determination of plasma drug concentrations up to 48 h after administration, as the drug concentrations in plasma were sufficiently high. As a result, all the pharmacokinetic parameters, including the elimination half-life, could be assessed reliably (Brutsche et al., 2000). We evaluate pulmonary equivalence using a crossover design in healthy subjects, which is more accurate; the CVs of the major pharmacokinetics parameter C_max_、AUC_0-t_ and AUC_0-∞_ were 22.69%,14.84% and 14.83%, correspondingly.

Our study shows that the average t_1/2_ (h) of the test formulation and reference formulation is about 11.09 h and 11.59 h which is similar as the data reported previously (>10 h after inhalation, a slower terminal elimination half-life after inhalation than after intravenous administration which is 7–8 h) (Derendorf et al., 1998; Mackie et al., 1996).The wash-out period is set as 5 days, which is more than seven times the half-life of the drug. The concentration of all subjects at time 0 of the two cycles was BLQ (below lower limit of quantification), and the lower limit of quantification provided by the testing party was 2.00 pg/mL. C_max_ is 239.83 ± 82.64 pg/mL, and BLQ is about 1% of C_max_. So the detection limit and cleaning period are set reasonably. The blood collection time should have three to five elimination half-life, or last until the blood concentration is 1/20–1/10 of C_max_. The t_1/2_ of this test is about 11.09 h, and the blood collection time lasts to 48 h, meeting the three to five half-life (31.83 h–53.10 h). The T_max_ of fluticasone propionate was 0.83 h for the test product, slightly later than for the reference products (0.66 h),so the blood collection time is set reasonably.

In this study. The results showed that there were no safety concerns during the study, and fluticasone propionate concentrations were similar after administration of the test and the reference product. The criteria for the BE was met, which are bioequivalent in terms of the rate and absorption.

## Data Availability

The original contributions presented in the study are included in the article/supplementary material, further inquiries can be directed to the corresponding authors.
